# Exploration of Predictive Factors for Acute Radiotherapy-Induced Gastro-Intestinal Symptoms in Prostate Cancer Patients

**DOI:** 10.3390/cancers17244035

**Published:** 2025-12-18

**Authors:** Pauline De Bruyn, Malgorzata Klass, Alain Van Muylem, Nicolas Jullian, François-Xavier Otte, Romain Diamand, Jean-Charles Preiser

**Affiliations:** 1 Radiation Oncology Department, Institut Jules Bordet, Hôpital Universitaire de Bruxelles (H.U.B.), Université Libre de Bruxelles (U.L.B.), Rue Meylemeersch 90, 1070 Brussels, Belgium; nicolas.jullian@hubruxelles.be (N.J.); francois-xavier.otte@hubruxelles.be (F.-X.O.); 2 Research Unit in Cardio-Respiratory Physiology, Exercise & Nutrition, Faculty of Human Movement Sciences, Université Libre de Bruxelles (U.L.B), 1070 Brussels, Belgium; malgorzata.klass@ulb.be; 3 Pneumology Department, Erasme, Hôpital Universitaire de Bruxelles (H.U.B.), Université libre de Bruxelles (U.L.B.), Route de Lennik 808, 1070 Brussels, Belgium; alain.vanmuylem@hubruxelles.be; 4 Urology Department, Institut Jules Bordet, Hôpital Universitaire de Bruxelles (H.U.B.), Université Libre de Bruxelles (U.L.B.), Rue Meylemeersch 90, 1070 Brussels, Belgium; romain.diamand@hubruxelles.be; 5 Department of Internal Medicine, Institut Jules Bordet, Hôpital Universitaire de Bruxelles (H.U.B.), Université Libre de Bruxelles (U.L.B.), Rue Meylemeersch 90, 1070 Brussels, Belgium; jean-charles.preiser@hubruxelles.be

**Keywords:** prostate cancer, pelvic radiotherapy, radiation-induced gastrointestinal symptoms, quality of life, predictive factors, systemic immune-inflammation index (SII), Diet Quality Index (DQI), obesity

## Abstract

**Background**: Acute gastrointestinal (GI) toxicity is a frequent adverse effect of pelvic radiotherapy (RT) in prostate cancer and predicts chronic complications. Identifying predictive factors, especially modifiable ones, is essential to guide supportive interventions. **Methods**: This prospective observational non-randomized cohort included 32 patients with prostate cancer treated with pelvic RT. Patient-reported GI symptoms were measured using the EORTC QLQ-PRT20 module, and clinician-reported toxicity was graded with CTCAE v5.0. Associations between GI outcomes and clinical, demographic, and lifestyle variables—including the systemic immune-inflammation index (SII) and the Diet Quality Index (DQI)—were examined using uni- and multivariable models. **Results**: Ninety-one percent of patients reported worsening GI symptoms during RT (median QLQ-PRT20 score increased from 4.2 to 26.8, *p* < 0.0001). In our final model, higher SII values were independently associated with greater symptom worsening (*p* = 0.001), whereas obesity (*p* = 0.03) and higher diet quality (*p* = 0.015) were protective. No significant interactions were found between SII and BMI or DQI, although diet quality partially attenuated the association between SII and symptom progression. Clinician-reported grade ≥ 2 GI toxicity occurred in 41% of patients and was significantly less frequent in obese individuals (adjusted OR = 0.04, 95% CI 0.0009–0.57, *p* = 0.02), with higher SII tending to increase risk and higher DQI showing a protective trend. **Conclusions**: In this exploratory analysis, systemic inflammation was associated with increased GI symptom burden, whereas obesity appeared to mitigate both patient- and clinician-reported outcomes. Higher dietary quality was similarly protective for patient-reported symptoms and showed a non-significant protective trend for clinician-reported toxicity. These findings highlight the interplay between metabolic and inflammatory status in shaping RT-related GI outcomes and support integrating nutritional and inflammatory profiling to guide personalized preventive strategies.

## Simple Summary

Radiotherapy (RT) is a standard treatment for prostate cancer but often induces acute gastrointestinal (GI) symptoms that can impair patients’ quality of life. We explored clinical and lifestyle-related factors that may predict acute GI patient reported symptoms and physician reported toxicity after RT. Higher systemic inflammation was linked to worse GI outcomes, whereas better diet quality and obesity were associated with lower symptom burden and toxicity rates. The apparent protective effect of obesity may result from anatomical factors, such as increased pelvic adiposity, that reduce radiation exposure to bowel structures. These findings suggest that integrating clinical, biological, and lifestyle data could help tailor supportive care strategies to improve the tolerance of RT in prostate cancer patients.

## 1. Introduction

Prostate cancer (PCa) is the most common cancer among men in Europe, with nearly 473,000 new cases reported in 2020 [1]. Radiotherapy (RT) is a standard treatment, achieving around 85–90% local and biological control at 5 years [2,3]. Despite technological improvements regarding RT conformality and normal tissue sparing as the use of volumetric modulated arc therapy (VMAT) [4,5,6], acute gastrointestinal (GI) toxicity (such as diarrhea, rectal and/or bowel pain, tenesmus, urgency, etc.) remain common, ranging from 3 to 30.8% grade ≥ 2 GI toxicity [5]. Although interruptions due to toxicities are uncommon in prostate RT, evidence shows that treatment gaps or prolonged overall treatment time can adversely affect clinical outcomes, including biochemical control in high-risk prostate cancer treated with protons and survival or local control in cervical and rectal cancers [7,8,9]. Acute symptoms are clinically relevant not only because of their immediate impact but also because they are predictors of chronic toxicity [10,11,12,13], which can persist for years, has limited therapeutic options, and substantially impairs quality of life (QoL) [14,15,16].

Patient-reported outcomes (PROs) provide unique insights into symptom burden and are still underrepresented [17]. Indeed, current prediction models for prostate RT toxicity are largely based on clinician-reported outcomes (CROs) and dosimetric features, whereas PROs and lifestyle variables remain rarely and inconsistently used as predictors [18]. The European Organization for Research and Treatment of Cancer (EORTC) recently developed the QLQ-PRT20 module [19], a validated questionnaire specifically designed to assess patient-reported GI symptoms associated with pelvic RT to be administered in addition to their core questionnaire (EORTC QLQ-C30). Coupled with CROs, such as CTCAE (Common Terminology Criteria for Adverse Events) toxicity grading [20], PROs allow for comprehensive evaluation of treatment-related adverse events [21].

To optimize patient management and prevent the impact of RT on QoL, identifying modifiable predictive factors might help. Several patient-related factors are known to influence the risk of RT-induced toxicity, including smoking, previous prostatectomy, diabetes, inflammatory bowel disease, and low body mass index (BMI) [22,23]. However, many potential predictors, such as diet quality [24,25,26], systemic inflammation [27,28,29], sarcopenia [30,31,32], and physical activity [33], remain underexplored. These factors, through their interplay with each other, gut microbiota [34,35,36,37], and local immune, inflammatory, and vascular responses to injury [38,39,40], may influence gut tolerance to RT. As systemic inflammation is known to be associated with obesity [41,42] and diet quality [43,44], the interaction between those predictors warrants particular attention.

This study aimed to identify clinical, biological, and lifestyle factors associated with acute RT-induced GI symptoms in patients with PCa. We hypothesized that both PROs and CROs would reveal modifiable predictors.

## 2. Materials and Methods

### 2.1. Study Population

This study was designed as a prospective observational non-randomized clinical cohort. From October 2023 to February 2024, 41 consecutive patients with localized or locally advanced prostate cancer scheduled for pelvic RT at the Institut Jules Bordet were screened for eligibility. Inclusion criteria were age ≥ 18 years, ECOG (Eastern Cooperative Oncology Group) performance status 0–2, and ability to complete questionnaires in French, Dutch, or English. Patients with prior pelvic RT, inflammatory bowel disease, celiac disease, or severe baseline GI symptoms (≥grade 3, CTCAE v5.0) were excluded. This study has been approved by the ethics committee of the Institut Jules Bordet with the reference number CE3650 and was registered on Clinicaltrial.gov under the ID NCT05880446.

### 2.2. Treatment and Follow-Up

Before simulation and treatment, all patients followed institutional bladder and bowel preparation protocols to ensure a comfortably full bladder (voiding 1 h before simulation or treatment followed by ingestion of 350 mL of water [45]) and an empty rectum (enema before simulation and glycerin suppository before treatment). Patients treated in the primary setting underwent placement of radiopaque fiducials markers one week before planning CT scan. MRI and/or CT-PET, when available, were co-registered with planning CT scan to help delineation.

Both prostate/prostate bed only RT (PORT) and whole-pelvis RT (WPRT) were represented. Clinical target volumes (CTV) were delineated on CT simulation following the ESTRO-ACROP guidelines for the prostate and prostate bed [46,47]. A 6 mm isotropic margin was applied to generate the PTV. Elective nodal regions included the internal, external, and common iliac chains (up to the aortic bifurcation), as well as the presacral and obturator regions. Nodal PTVs were created by applying a 7 mm isotropic expansion to the nodal CTV. Patients received 60 Gy in 20 fractions to the prostate or 55 Gy in 20 fractions to the prostate bed, with 47 Gy delivered to elective pelvic lymph nodes when WPRT was performed. A simultaneous integrated boost (SIB) to the prostate or to PET-positive pelvic lymph nodes was allowed using co-registered imaging, provided that all OAR constraints were respected.

Organs at risk (OARs)—including the bladder, rectum, sigmoid colon, small bowel, penile bulb, femoral heads, and prostatic urethra when visualized—were delineated, and dose constraints were applied mainly according to the DELINEATE trial and the POP-RT toxicity analysis [48,49]. A Planning organ at Risk Volume (PRV) of 5 mm was added to the rectum, except for anteriorly, and a PRV of 3 mm was applied to the sigmoid colon (defined as the bowel portion from the rectum to the right iliac fossa) and to the bowel loops. Mandatory rectal constraints were D02 < 61.6 Gy, V60.8 < 5%, V56.8 < 15%, V52.7 < 30%, V48.7 < 50%, and V40.5 < 60%. For the sigmoid colon and for the bowel loops, mandatory constraints were Dmax < 52.7 Gy, V48.7 < 6 cc, V44.6 < 28 cc, V40.5 < 110 cc, and V36.5 < 158 cc.

Patients were positioned supine with their arms on their chest and stabilized using knee and foot supports. Treatments were delivered using volumetric modulated arc therapy (VMAT) on Elekta Versa HD and Elekta Harmony linear accelerators with 6-MV photon beams. All patients underwent daily Cone-Beam Computed Tomography (CBCT)-based image-guided radiotherapy (IGRT), with or without concomitant androgen deprivation therapy (ADT).

Patients were followed from baseline consultation to completion of RT. Supportive antidiarrheal medication was offered when grade ≥ 2 gastrointestinal toxicity was documented by the treating clinician during or after treatment. Pre- and/or probiotics were not offered during RT.

### 2.3. Outcomes

Primary PRO: Change in GI symptoms during RT, defined as the difference in GI symptom score between T1 (end of RT) and T0 (baseline, before RT) and measured using the EORTC QLQ-PRT20 module [13]. For this study, an exploratory global GI symptom score was created at each timepoint by computing the mean of the linearly transformed scores of all QLQ-PRT20 subscales. Higher scores indicate worse GI symptoms; therefore, a positive difference (T1–T0) reflects a worsening of symptoms during RT. The overall QoL was also measured using the EORTC core questionnaire (QLQ-C30) and computed according to the EORTC guidelines for scoring of the QLQ-C30 Summary Score. Higher scores indicate a better QoL; therefore, a negative difference (T1–T0) reflects a worsening of overall QoL during RT.

Secondary CRO: Clinician-reported GI toxicity, graded using CTCAE v5.0 [20] at the end of RT, dichotomized as <grade 2 vs. ≥grade 2.

### 2.4. Candidate Predictors

Clinical and lifestyle variables included age, BMI (kg/m^2^), tobacco exposure (never exposed vs. previously or currently exposed), alcohol consumption (within vs. exceeding recommendations), physical activity (steps) [50], constipation history (yes/no), waist circumference (cm), Skeletal Muscle Index (SMI) to determine CT-defined sarcopenia (yes/no) [51,52], systemic immune-inflammation index (SII) [28,53], Diet Quality Index (DQI) [54], prior prostatectomy (yes/no), and RT field size (PORT vs. WPRT).

BMI was categorized as normal (<25 kg/m^2^), overweight (25–29.9 kg/m^2^), and obese (≥30 kg/m^2^). Alcohol consumption was categorized according to CSS guidelines, which define the recommended limit as ≤10 standard units per week (1 unit = 10 g of alcohol) [55]. Physical activity was represented by the daily average number of steps measured over a two-week period using a wrist-worn tracker (Polar Ignite (2019), Polar^®^, Kempele, Finland) immediately before the start of RT. The variable was then categorized into three subgroups—inactive (<5159 steps/day), low to moderately active (5159–9914 steps/day) and active (≥9915 steps/day)—corresponding to the tertiles of the continuous variable. These thresholds were used because they are close to those defined in the Graduated Step Index classification [56] and allowed for a balanced distribution of participants across subgroups. Waist circumference was classified into low to moderate (<102 cm) and high (≥102 cm) cardio-vascular (CV) risk categories. The SMI was measured on simulation CT scans using RT delineation software MIM^®^ (MIM Software, Inc., Version 7.4.2., Cleveland, OH, USA) following a recently validated method developed for this purpose (publication ongoing). CT-defined sarcopenia (yes/no) was determined according to literature-based SMI cut-offs adjusted for sex and BMI [57]. The SII was computed from a pre-treatment blood sample collected within three months before the start of RT. This time window was chosen to ensure availability of complete blood counts while excluding measurements obtained during any documented acute inflammatory event, infection, or initiation of a new medication that could influence leukocyte or platelet counts. For patients receiving ADT, SII measurements were obtained before or at the time of ADT initiation when available; however, for those treated with neoadjuvant ADT, SII necessarily reflected values under ongoing ADT. The SII was calculated using the formula SII = platelet count × neutrophil count/lymphocyte count and dichotomized into low vs. high risk of adverse oncological outcomes according to a literature-based median cut-off (576) [53]. Dietary habits were assessed using a 1-week food diary and a structured diet history conducted by a medical nutritionist prior to RT start. Dietary data were analyzed with NubelPro^®^ dietary planning platform (Nubel^®^, Brussels, Belgium; database updated February 2022, accessed in 2025), and diet quality was quantified using the Diet Quality Index-Revised (DQI-R) scale [54], ranging from 0 to 100. Clinical categorization of DQI scores was proposed to differentiate patients with a low (<55), moderate (55–74), or high (≥75) diet quality.

### 2.5. Statistical Analysis

All analyses were conducted using R Studio Version 2023.12.0+369 with R Version 4.3.2 [58]. Descriptive statistics were used to summarize patient characteristics.

For PROs (primary continuous outcome), after normalization, we used the F-test from ANOVA to compare the mean transformed scores between subgroups of categorical variables. Then, univariate and multivariate linear regression analyses were used to explore the associations between the potential predictors and patient-reported GI symptoms. Potential interactions between explanatory variables were explored by introducing them as interactive terms into multivariable models. If this introduction lowered the global quality of our model, we performed descriptive, univariate, and specific multivariate analyses to explore the association between the outcome and the interactive terms only.

For CROs (secondary binary outcome), after dichotomization of the dependent variable, we used a Chi-square test (or Fisher’s exact test if small values were expected) to compare the prevalence of the categorical variables. Then, we used univariate and multivariate logistic regression to estimate the association of these variables with clinician-assessed GI toxicity. If some predictors showed complete separation in the data, causing the multivariable model not to converge and the estimated odds ratios to become unreliable, we applied penalized logistic regression and simplified the model.

For multivariable modeling of both PROs and CROs, the initial set of candidate predictors was defined a priori based on clinical relevance. Among these variables, those with a univariate *p* < 0.20 were retained, and data-driven procedures (backward stepwise and best-subset selection) were applied only to refine this clinically preselected pool and improve model parsimony. All statistical tests were two-sided, and a *p* < 0.05 was considered statistically significant. Given the exploratory nature and small sample size, emphasis was placed on effect sizes and clinical interpretation rather than strict statistical significance.

## 3. Results

This cohort included thirty-two patients; the main endpoint was the progression of patient-reported GI symptoms during RT, and the secondary endpoint was the incidence of clinician-reported grade ≥ 2 GI toxicity.

### 3.1. Patient Characteristics

**Figure 1:**
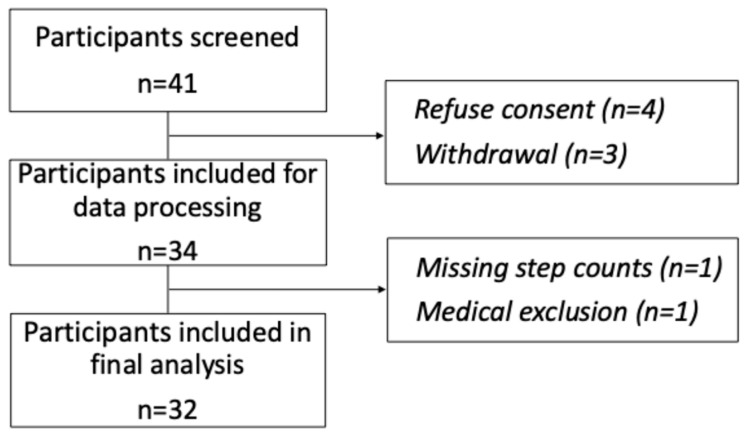
Flow chart of participant inclusion and analysis.

Of the 41 selected patients based on inclusion criteria, 34 consented, and 33 had complete data. One additional patient was excluded prior to analysis due to the incidental finding of a marked acute exacerbation of fluctuating chronic thrombocytopenia, most likely related to immune thrombocytopenic purpura (ITP), which occurred during RT and required treatment with corticosteroids and intravenous immunoglobulins. (Figure 1). Median age was 72 years (IQR 65–77). Nearly half had prior prostatectomy (52%) and 85% underwent WPRT. Most patients received concomitant ADT (91%), including four patients who had initiated ADT neoadjuvantly. Overweight and obesity were common (82%). Baseline characteristics are shown in Table 1.

### 3.2. Patient-Reported Outcomes

**Figure 2:**
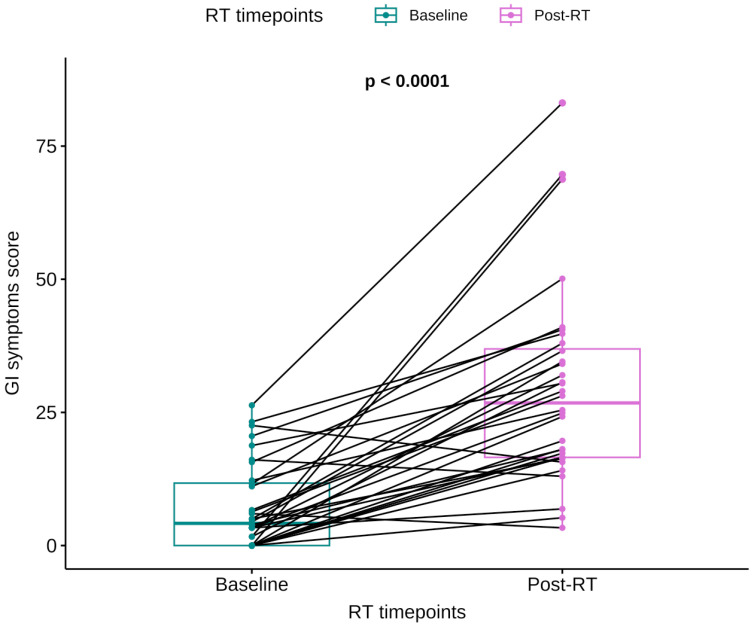
Representation of individual and median change of PRT20 score between the start and the end of RT.

Ninety-one percent (91%) of patients reported worsening of GI symptoms during RT, with median QLQ-PRT20 scores increasing from 4.17 (IQR 11.7) at baseline to 26.8 (IQR 20.4) at the end of treatment (Wilcoxon signed-rank test; *p* < 0.0001) (Figure 2).

In parallel, overall QoL significantly worsened during RT in 88% of patients, with a median QLQ-C30 total score decreasing from 92.7 (IQR 9.53) at baseline to 80.2 (IQR 15.3) at the end of RT (Wilcoxon signed-rank test; *p* < 0.0001).

After normalization (Box-Cox transformation) of the GI symptoms score difference to satisfy modeling assumptions, univariable linear regressions showed that patients with higher systemic immune-inflammation index (SII) experienced significantly greater worsening of GI symptoms (*p* = 0.005), whereas higher dietary quality (DQI) was associated with smaller increases in symptom scores (*p* = 0.002). A history of constipation was also significantly associated with less pronounced symptom worsening (*p* = 0.02). A borderline association was observed for RT field, with patients receiving whole-pelvis RT showing higher mean symptom increase compared with those treated with prostate-only RT (*p* = 0.053). No other clinical or lifestyle variables were significantly related to changes in the normalized GI symptom score. (Table 2).

In our multivariable linear regression model, including clinical and lifestyle factors, the transformed difference in GI symptom score was significantly explained by the overall model (adjusted R^2^ = 0.54, *p* = 0.0016). This model showed that holding all other variables constant, obesity was associated with lower symptom score progression (b = −4.8, *p* = 0.015), whereas higher SII values were linked to increased score progression (b = 0.008, *p* = 0.018). A high dietary quality index showed a non-significant trend toward lower symptom scores (b = −4.7, *p* = 0.097). No significant associations were observed for smoking, constipation, prostate cancer history, concomitant ADT, or radiotherapy field (Table 3).

To account for baseline variability, an ANCOVA model was used with the end-of-treatment PRT20 score as the dependent variable and baseline score as a covariate. In this model, obesity remained associated with lower adjusted symptom scores compared with normal BMI (*p* = 0.046), while high diet quality was also associated with lower adjusted end-of-treatment symptoms (*p* = 0.042). Higher SII remained significantly associated with worse symptoms (β = 0.00165 per unit, 95% CI 0.00024–0.00306; *p* = 0.024).

Adjusted marginal mean differences were then estimated. The direction of the pairwise contrasts was consistent with the ANCOVA coefficients; however, due to the small sample size and the use of Tukey correction for multiple comparisons, none of the group-to-group contrasts reached statistical significance (e.g., obese vs. normal: adjusted mean difference—0.88, 95% CI −1.78 to 0.02; *p* = 0.11). As expected, this correction yields wider confidence intervals and more conservative *p*-values than the regression coefficients.

Importantly, the association between constipation history and reduced symptom worsening observed in the univariate change-score analysis was no longer significant in the ANCOVA model (*p* = 0.17), indicating that this apparent effect was largely driven by higher baseline symptom levels rather than a true protective association.

Because several variables showed limited contribution in the full model and to improve model parsimony and stability, a second multivariable model was built using stepwise and best-subset selection methods. In this reduced model–including only predictors with the strongest explanatory value (BMI category, DQI and SII)—the transformed difference in GI symptom score was significantly explained by obesity, dietary quality, and systemic inflammation (adjusted R^2^ = 0.57, *p* < 0.001). Obesity was associated with lower symptom scores (b = −3.6, *p* = 0.031), while higher SII values were linked to increased scores (b = 0.01, *p* = 0.001). In this model, a high dietary quality index was also significantly related to lower symptom scores (b = −6.0, *p* = 0.015) (Table 4). Although the normality assumption was not fully met (Shapiro–Wilk *p* = 0.014), this affects only the precision of *p*-values but not the direction or magnitude of the estimated coefficients. Given the robustness of linear regression to moderate non-normality, the estimated coefficients were considered reliable, although *p*-values should be interpreted with caution.

### 3.3. Interactions in the Relationship Between Systemic Inflammation and Patient-Reported GI Symptoms

#### 3.3.1. Interaction with BMI Categories

**Figure 3:**
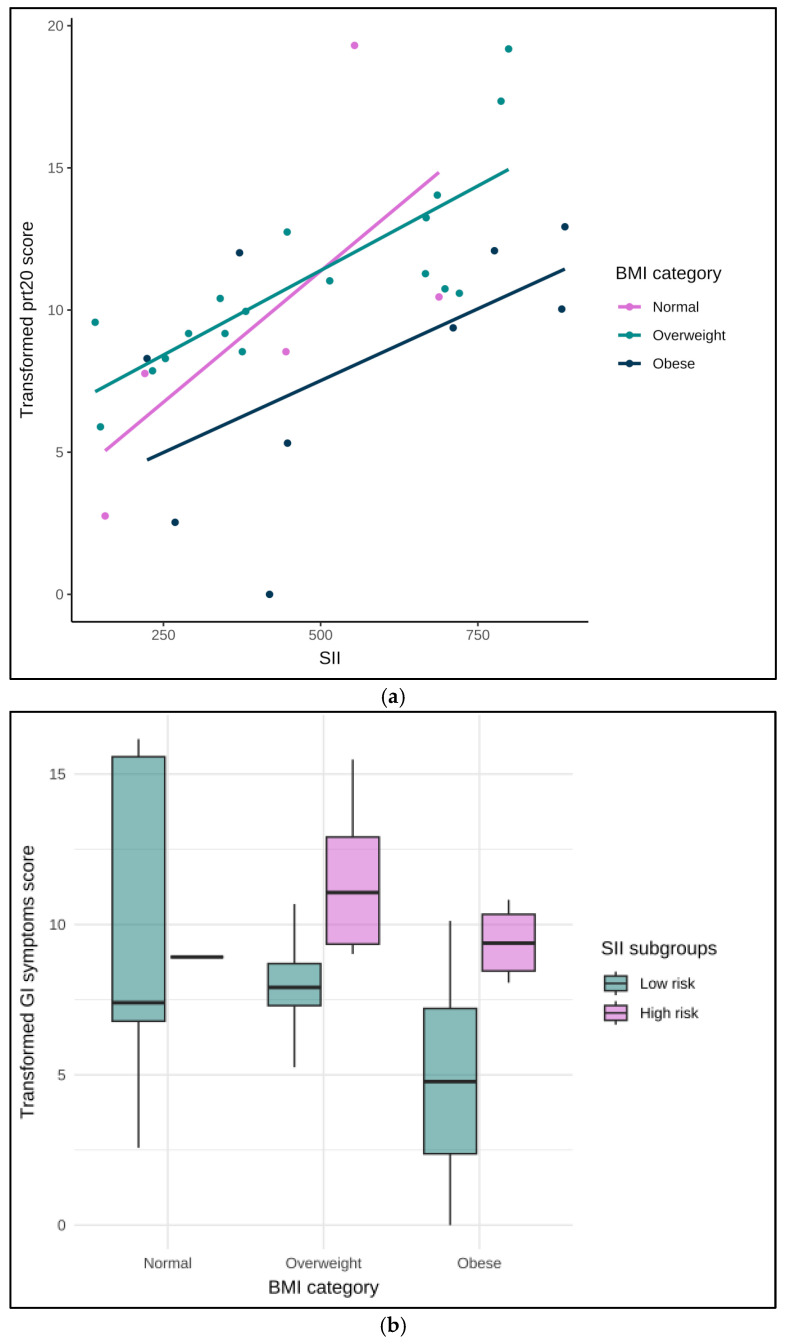
(**a**) Association between the systemic immune-inflammation index (SII) and the transformed GI symptoms score, stratified by BMI category (normal (n = 5), overweight (n = 18, obese (n = 9)). Lines indicate linear fits within each subgroup. BMI: body mass index; GI: Gastro-Intestinal; SII: systemic immune-inflammation index. (**b**). Transformed gastrointestinal (GI) symptom score across BMI categories (normal, overweight, obese), stratified by systemic immune-inflammation index (SII) subgroups (low (n = 20) vs. high (n = 12) risk of oncological outcomes). Boxplots display median, interquartile range, and variability within each group. BMI: body mass index; GI: Gastro-Intestinal; SII: systemic immune-inflammation index.

We introduced BMI as an interactive term in the multivariable linear regression and found no interaction between BMI and SII. Because the addition of this categorial variable (normal, overweight vs. obese) lowered the global quality of our model, we also assessed this interaction in the second model (less variables), performed univariate and descriptive analyses of the direct association between BMI and SII in their various forms (continuous or categorial), and found no significant association (Figure 3a).

Indeed, when observing the relationship between patient-reported GI symptoms and BMI categories according to systemic inflammation status, the high SII subgroup seemed to have a greater change in GI symptom score than lower SII subgroups within each BMI category (Figure 3b).

#### 3.3.2. Interaction with Diet Quality

**Figure 4:**
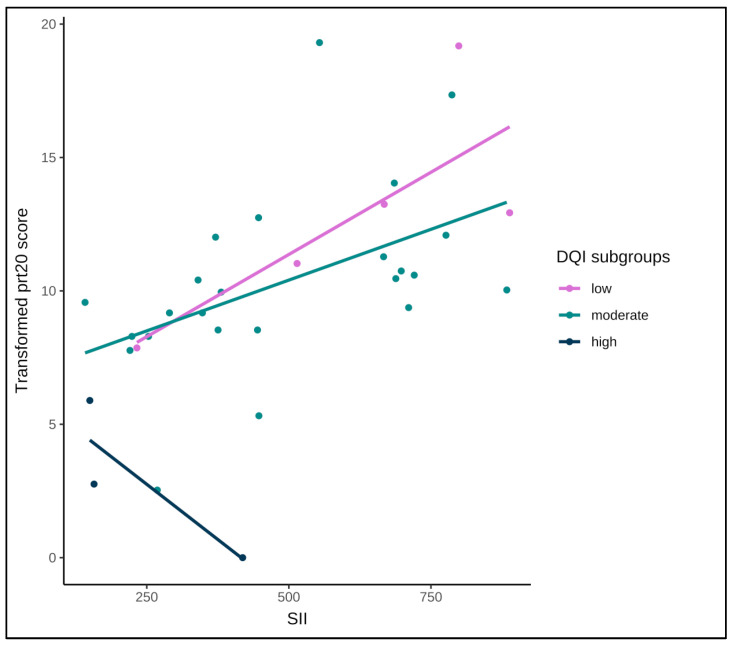
Association between the systemic immune-inflammation index (SII) and the transformed GI symptom score, stratified by dietary quality index (DQI) subgroups (low (n = 5), moderate (n = 24), high (n = 3)). Lines indicate linear fits within each subgroup. DQI: Diet Quality Index; GI: Gastro-Intestinal; SII: systemic immune-inflammation index.

The interaction between SII and DQI was not statistically significant (*p* = 0.24), despite the visual impression (Figure 4) suggesting a possible difference in the relationship between SII and GI symptom score progression across DQI subgroups. The estimated slopes indicated a positive association between SII and symptom worsening in the low (b = 0.012) and moderate (b = 0.007) DQI groups but a flat or slightly negative trend in the high DQI group (b = −0.005). However, these differences were not significant in pairwise comparisons (all *p* > 0.25), likely due to the small number of participants in the high DQI subgroup and overlapping confidence intervals.

Nevertheless, using the second model to assess this interaction, we observed that both SII (*p* < 0.001) and DQI level (*p* = 0.016) remained independently associated with GI symptom score progression (as well as BMI category, *p* = 0.031). Model diagnostics indicated a deviation from normality, meaning once again that *p*-values should be interpreted with caution.

Knowing that DQI showed a trend toward direct association with SII (*p* = 0.077) and was independently related to GI symptom scores, we also tested its potential confounding effect. When DQI was added to the multivariable model, the coefficient for SII decreased by approximately 22%, suggesting a possible confounding pattern in the relationship between SII and GI symptom progression during RT. However, given the limited sample size and the deviation from model normality, this analysis remains exploratory; no formal mediation or confounding analysis was performed, and this potential confounding pattern should be interpreted with caution.

### 3.4. Clinician-Reported Toxicity

Grade ≥ 2 GI toxicity was observed in 41% of patients. The prevalence of ≥grade 2 toxicity was not significantly different in any subgroups of categorical variables. As continuous variables, only the mean BMI was statistically significantly lower in the subgroup of patients with grade ≥ 2 GI toxicity (*p* < 0.05), and the mean DQI was slightly lower in the high-toxicity subgroup, but this difference was not significant (*p* = 0.087).

In univariate analyses, none of the clinical or lifestyle factors were significantly associated with the occurrence of grade ≥ 2 GI toxicity. A borderline association was observed for BMI categories (global *p* = 0.07), with lower odds of toxicity in obese patients compared to normal-weight individuals (OR = 0.08, 95% CI 0.00–1.03, *p* = 0.08). Although the global likelihood ratio test (LRT) suggested an overall association between DQI and GI toxicity (*p* = 0.04), this result should be interpreted cautiously. None of the individual DQI categories reached significance on Wald tests, and no toxicity events occurred among patients with high DQI, indicating potential sparse data bias and unstable estimates (Table 5).

In the full multivariable model, obesity was the only variable significantly associated with reduced odds of grade ≥ 2 GI toxicity (OR = 0.02, *p* = 0.025), while higher dietary quality showed a borderline protective trend (OR = 0.01, *p* = 0.07). However, the overall model was not significant, and estimates should be interpreted cautiously given the small sample size and sparse data in some subgroups.

To improve model stability and interpretability, a reduced penalized logistic regression model including BMI category, dietary quality, and systemic inflammation was built. This model significantly predicted GI toxicity (LRT *p* = 0.044).

In this second model, obesity was independently associated with a markedly lower likelihood of grade ≥ 2 GI toxicity (adjusted OR = 0.04, 95% CI 0.0009–0.57, *p* = 0.02; global *p* = 0.08). Higher systemic inflammation tended to increase the risk of toxicity (adjusted OR = 9.75, 95% CI 0.64–27.5, *p* = 0.15; global *p* = 0.06), whereas better dietary quality showed a non-significant trend toward a protective effect (adjusted OR = 0.04, 95% CI 0.0002–1.27, *p* = 0.07; global *p* = 0.29). Although none of the variables reached formal global significance, the direction and magnitude of effects suggest that both obesity and higher diet quality may confer protection against treatment-related GI toxicity, while elevated systemic inflammation could contribute to increased risk. (Table 6).

### 3.5. Integration of Outcomes

Both PROs and CROs consistently identified obesity as a protective factor against GI side effects. Systemic inflammation was associated with worse PROs and showed a concordant trend toward higher clinician-reported toxicity. Higher dietary quality emerged as a protective factor for patient-reported symptoms and showed a similar trend for clinician-reported toxicity. No significant interaction between SII and either BMI or DQI was found, although higher diet quality might partly attenuate the association between inflammation and symptom worsening. These findings highlight the complementary insights provided by patient- and clinician-reported measures and suggest a convergent role of metabolic and inflammatory status in modulating treatment-related GI toxicity.

## 4. Discussion

### 4.1. Key Findings

This prospective study confirms that acute GI toxicity is frequent in patients with prostate cancer receiving pelvic RT. Both patient- and clinician-reported outcomes suggested a coherent pattern of clinical and lifestyle-related factors associated with symptom progression. In this small cohort, higher systemic inflammation was consistently associated with greater worsening of GI symptoms, while higher dietary quality was associated with lower symptom progression and showed a similar, though non-significant, protective trend for clinician-reported toxicity. Obesity emerged as a potential protective factor in both outcome domains, including a significantly lower odds of ≥grade 2 toxicity; however, this finding should be interpreted cautiously given the small sample size and the exploratory nature of the analysis. Overall, these results suggest that metabolic and inflammatory status may jointly influence individual susceptibility to radiation-induced GI side effects.

### 4.2. Clinical Interpretation

The significant increase in patient-reported GI symptoms observed supports the clinical impression that acute GI toxicity remains a frequent side effect of pelvic RT despite modern treatment techniques. This emphasizes the urgent need to explore and identify modifiable factors to optimize patient management in a preventive manner.

Obesity is known to be associated with chronic inflammation [41,42], but its impact on RT GI side effects is still not clear [23,59]. In our study, systemic inflammation, approximated by the variable SII, was associated with worse outcomes; therefore, obesity would have been expected to be an adverse predictor. Interestingly, we observed the opposite: obesity appeared protective for patient-related RT-induced GI symptoms.

The paradoxical protective effect of obesity in our sample could be explained by anatomical “spacing” due to differences in pelvic adiposity, which could increase the distance between bowel loops and high-dose regions. Because patient-specific dosimetric data on bowel exposure were not collected in this study, we cannot determine whether such anatomical spacing contributed to the associations observed. Future work should incorporate detailed post-treatment bowel-delivered dose metrics to clarify whether GI symptoms are more closely related to actual bowel dose than BMI category per se.

Moreover, given our small sample size and the resulting reduced statistical power, this association with obesity must be interpreted with caution. This finding certainly does not imply that weight gain prior to RT should be encouraged but rather that greater attention to dose constraints may be warranted for patients with normal weight and less abdominal fat.

On the other hand, abdominal adiposity has also been associated with increased intrafraction prostate motion, possibly driven by more pronounced respiratory-induced pelvic movement in obese patients [60]. Such motion may, in principle, require larger PTV margins to maintain adequate target coverage, which would increase the volume of rectum and sigmoid exposed to intermediate to high doses and thereby elevate the risk of GI toxicity. This mechanism acts in the opposite direction of the anatomical “spacing” hypothesis described above and highlights that the net effect of obesity on pelvic dose distribution is not straightforward. In our cohort, PTV margins were not adapted for BMI, and actual target coverage or motion patterns were not verified retrospectively, preventing us from assessing whether either mechanism dominated in practice.

Future studies should quantify and incorporate the actual RT dose delivered to bowel structures to clarify whether the observed association between obesity and GI symptoms reflects true biological effects or differences in dose exposure. In parallel, evaluating intrafraction target and OAR displacements in these subgroups would help determine whether individualized PTV and PRV margins—tailored to pelvic morphology—may be warranted.

As a potential interaction may exist between obesity and systemic inflammation, we tested the interaction between those two factors in multiple ways and found no significant association. This suggests that, in our sample, overweight and obese patients may present with or without systemic inflammation and that according to our previous model, greater systemic inflammation is associated with greater increases in patient-reported GI symptoms after RT, independent of patient’s BMI category and vice versa.

Thus, SII emerges as a potential biomarker of increased toxicity risk. Indeed, it has already proven its predictive value in clinical outcomes such as biological recurrence after prostatectomy [29] but also toxicity, such as radiation cystitis in patients with gynecological cancer [28]. Its role as both a predictor and therapeutic target deserves further investigation.

Knowing that systemic inflammation might be influenced by diet quality [43,44], we further tested the interaction and confounding effect of the DQI on the association of SII with patient-reported GI symptoms during RT.

Although the interaction between dietary quality and systemic inflammation did not reach statistical significance, their consistent and independent associations with GI symptom progression suggest that these two factors may act through related biological pathways. The attenuation of the SII effect after inclusion of DQI supports potential partial confounding, indicating that better dietary quality might buffer the impact of pre-existing systemic inflammation on RT-induced mucosal injury and subsequent GI symptoms.

In other words, patients with higher diet quality or trained to improve their diet quality before treatment could experience improved tolerance to pelvic RT. However, the small number of participants with high DQI in this pilot study limits the robustness of this conclusion, and future larger cohorts are warranted to confirm this potentially protective role.

Nevertheless, this hypothetical protective effect of diet quality is consistent with the existing literature suggesting that nutritional modulation during or after RT might mitigate treatment side effects [61,62] and that microbiota—mainly influenced by diet—may shape inflammatory responses and intestinal barrier integrity during RT [34,35,36,37,63]. Indeed, a high-fiber diet is known to exert anti-inflammatory effects and improve gut barrier function through enhancement of microbiota diversity and promotion of beneficial bacteria’s growth [24,64,65]. Because our study did not include microbiota measurements, these mechanistic considerations cannot be evaluated within our cohort and should be regarded as speculative, referring exclusively to established evidence from the broader literature. Future studies should analyze microbiota composition alongside diet quality to better elucidate the mechanisms underlying its interaction with local, systemic inflammation and RT GI symptoms and to clarify whether such pathways contribute to the patterns observed in this study. In addition, if stool samples can be collected, analysis of other inflammatory biomarkers, such as fecal calprotectin, could help clarify the relationship between diet quality and RT-induced GI symptoms. Indeed, fecal calprotectin is a non-invasive marker of gut inflammation commonly used to diagnose and monitor therapeutic response in inflammatory bowel disease and has been associated with acute RT-induced GI toxicity [66,67,68].

Finally, we did not observe an association between sarcopenia or low muscle mass on CT—present in 47% of our cohort—and increased GI symptoms or GI toxicity, despite prior concerns that reduced muscle mass may impair RT tolerance [51,52]. Similar null associations have also been reported in other pelvic malignancies, such as bladder cancer [69], whereas studies in rectal and gynecological cancers have more frequently described less favorable outcomes in sarcopenic patients [70,71,72]. Our findings align with recent work by Vickers et al. [73], who similarly reported that sarcopenia did not predict acute or late clinician-reported toxicities in PCa patients treated with RT, although PROs were not assessed. It is worth noting that contemporary definitions of sarcopenia, such as those proposed by the EWGSOP2 and the Sarcopenia Definitions and Outcomes Consortium (SDOC), show limited agreement [74], and both require direct measures of muscle strength (e.g., handgrip dynamometry [75]). We therefore encourage future studies to combine SMI assessment with functional strength measures to better characterize the relationship between clinical sarcopenia and pelvic RT tolerance.

### 4.3. Clinical Implications

Our findings suggest potential avenues for more personalized supportive care in patients undergoing pelvic RT. The SII, which is easily derived from routine blood tests, may help identify individuals at higher risk of symptom worsening and thus guide targeted preventive strategies. Dietary quality also emerged as a potentially modifiable factor associated with symptom progression, supporting the rationale for evaluating structured nutritional interventions in future trials.

Because the relationship between BMI and GI toxicity likely reflects competing anatomical and motion-related mechanisms, BMI itself should not be considered a modifiable clinical target; instead, future studies evaluating delivered dose and intrafraction motion may clarify whether specific pelvic morphologies warrant tailored dosimetric vigilance. Integrating these elements with microbiota analyses may enhance mechanistic understanding and improve risk stratification.

Finally, integration of PROs with CROs offers a more comprehensive assessment of treatment-related toxicity and may better inform both preventive and early management strategies.

### 4.4. Limitations

The main limitations are the single-center design and small sample size, which limit statistical power, precision, and generalizability. The strong imbalance in RT field groups (WPRT vs. PORT) and the high rate of ADT exposure further constrain subgroup analyses and confounding control. In particular, the very small number of patients treated with PORT precludes any reliable interpretation of field-related effects and likely contributed to the borderline association observed in univariable analysis; estimates derived from this subgroup should therefore be interpreted with caution. As a result, some known risk factors were not captured (e.g., concomitant ADT, RT field size). Residual confounding may also affect interpretations. For instance, diabetes—a known risk factor—was not represented in our sample and therefore was not included in our models. Additionally, for patients who initiated ADT neoadjuvantly, SII values were necessarily measured under ongoing ADT, which may have influenced inflammatory markers and introduced residual confounding.

Moreover, the actual RT dose delivered to bowel structures was not available for this analysis; this could have influenced, attenuated, or eliminated the observed positive association between BMI and GI toxicity/symptoms. Although all bowel dose constraints were respected, planned DVH metrics were not used as surrogates because they may inadequately reflect the dose truly received by mobile bowel loops and the deformable rectum [76,77]. A CBCT-based delivered-dose assessment is currently underway and will complement these exploratory findings. In addition, we did not assess intrafraction prostate motion or target coverage adequacy, which may interact with BMI and influence rectal and/or sigmoid colon dose exposure.

Given the small sample size, even clinically guided variable selection carries a risk of overfitting, reinforcing the exploratory nature of these findings. In addition, transforming continuous predictors into categories (e.g., DQI groups) may have led to information loss.

Finally, microbiota analyses were not performed in this exploratory study because of the cost, limiting our ability to interpret the observed interaction between diet quality and the association of systemic inflammation with GI symptoms. Larger cohorts with integrated clinical, dosimetric, microbiota, and biological data are needed to confirm these findings.

## 5. Conclusions

Acute GI toxicity remains common during pelvic RT for prostate cancer. In this cohort, both patient-reported and clinician-reported outcomes suggested that obesity may be associated with a lower risk of acute GI side effects, while higher systemic inflammation was associated with worse gastrointestinal outcomes. Better dietary quality also emerged as a protective factor, significantly mitigating symptom worsening and showing a similar trend toward reduced clinician-reported toxicity. Although no significant interactions were found, dietary quality appeared to partly attenuate the impact of systemic inflammation on treatment tolerance. These exploratory findings warrant further investigation of metabolic and inflammatory profiles, along with nutritional factors, to improve the prediction of and resilience to radiation-induced toxicity.

## Figures and Tables

**Table 1:** Baseline characteristics of the study population (*n* = 32).

Characteristics		
Age (years)		73 (65–78)
Tobacco exposure		
	Never exposed	17 (53%)
	Previously or currently exposed	15 (47%)
Alcohol consumption		
	Within recommendations	18 (56%)
	Exceed recommendations	14 (44%)
Daily step count (n)		7276 (4515–10,787)
Physical activity levels [daily step count]		
	Inactive [≤5158]	11 (34%)
	Low to moderately active [5159–9915]	10 (31%)
	Active [≥9916]	11 (34%)
Constipation		
	Known chronic constipation	9 (28%)
BMI (kg/m^2^)		28.0 (26.3–30.1)
BMI category		
	Normal [18.5–24.9]	5 (16%)
	Overweight [25.0–29.9]	18 (56%)
	Obese [≥30.0]	9 (28%)
Waist circumference [cm]		
	Low/medium CV risk [<102]	13 (41%)
	High CV risk [≥102]	19 (59%)
SMI (cm^2^/m^2^)		52 (46, 60)
Sarcopenia on CT		15 (47%)
SII		446 (279–693)
SII risk group		
	Low risk [<576]	20 (63%)
	High risk [≥576]	12 (38%)
DQI		65 (60–70)
DQI level		
	Low [≤55]	5 (16%)
	Moderate [56–75]	24 (75%)
	High [≥76]	3 (9.4%)
Previous prostatectomy		16 (50%)
Concomitant ADT		29 (91%)
RT field size		
	PORT	5 (16%)
	WPRT	27 (84%)

Values are median (Q1–Q3) or numbers (percentage), as appropriate. ADT: androgen deprivation therapy; BMI: body mass index; CT: Computed Tomography; CV: cardio-vascular; DQI: Diet Quality Index; PORT: prostate/prostate bed only RT; RT: Radiation Therapy; SII: systemic immune-inflammation index; SMI: Skeletal Muscle Index; WPRT: whole-pelvis RT.

**Table 2:** Mean GI symptom score difference of each subgroup of categorical predictors (±SD) and univariable linear regression of the association between all predictors and GI symptom score difference.

Predictor		Mean ± SD			Univariable b ± SE			*p*
	Categories (n)							
Total (32)		10.0 ± 4.21						
Tobacco exposure								0.083 '
	Non-exposed (17)	8.80 ± 4.11			Ref			
	Exposed (15)	11.4 ± 4.01			2.5 ± 1.4			
Alcohol consumption								0.09 ' ✣
	Within recommendations (18)	9.09 ± 3.89			Ref			
	Exceed recommendations (14)	11.2 ± 4.44			2.1 ± 1.5			
Constipation history								0.02 * ✣
	No (23)	11.0 ± 3.99			Ref			
	Yes (9)	7.48 ± 3.84			−3.5 ± 1.55			
BMI category								0.22
	Normal (5)	9.76 ± 6.04			Ref			
	Overweight (18)	11.1 ± 3.29			1.3 ± 2.1			(0.81)
	Obese (9)	8.06 ± 4.53			−1.7 ± 2.3			(0.74)
Waist circumference								0.94
	Low/medium CV risk (13)	10.1 ± 3.25			Ref			
	High CV risk (19)	9.97 ± 4.84			−0.1 ± 1.5			
Sarcopenia on CT								0.93
	No (17)	9.95 ± 4.31			Ref			
	Yes (15)	10.1 ± 4.24			0.14 ± 1.5			
Physical activity level								0.51
	Inactive (11)	8.83 ± 5.30			Ref			
	Low to moderately active (10)	10.4 ± 1.74			1.5 ± 1.9			
	Active (11)	10.9 ± 4.65			2.1 ± 1.8			
SII risk group								0.005 **
	Low risk (20)	8.46 ± 4.10			Ref			
	High risk (12)	12.6 ± 3.01			4.2 ± 1.4			
DQI level								0.002 **
	Low (5)	12.8 ± 4.14			Ref			
	Moderate (24)	10.3 ± 3.41			−2.5 ± 1.7			(0.32)
	High (3)	2.88 ± 2.95			−9.9 ± 2.6			(0.001) **
Previous prostatectomy								0.72
	No (16)	9.75 ± 5.04			Ref			
	Yes (16)	10.3 ± 3.32			0.5 ± 1.5			
Concomitant ADT								0.26
	No (3)	7.38 ± 1.79			Ref			
	Yes (29)	10.3 ± 4.31			2.9 ± 2.5			
RT field								0.053 '
	PORT (5)	6.69 ± 2.58			Ref			
	WPRT (27)	10.6 ± 4.19			3.9 ± 1.9			
Age					−0.006 ± 0.09			0.95
Step (n)					0.0001 ± 0.0002			0.57
BMI (kg/m^2^)					−0.2 ± 0.2			0.37
SMI (cm^2^/m^2^)					−0.01 ± 0.09			0.90
SII					0.01 ± 0.003			0.0004 ***
DQI					−0.16 ± 0.09			0.057 '

' *p* < 0.2, * *p* < 0.05, ** *p* < 0.01, *** *p* < 0.001; F-Test from ANOVA or non-parametric Kruskal–Wallis test according to normality of residuals for categorical variables. ✣ Assumption of normality of residuals not satisfied even with transformed dependent variable (but similar *p*-value with non-parametric Kruskal–Wallis test for categorical variables). ADT: androgen deprivation therapy; BMI: body mass index; CT: Computed Tomography; CV: cardio-vascular; DQI: Diet Quality Index; PORT: prostate/prostate bed only RT; RT: Radiation Therapy; SD: Standard Deviation; SE: Standard Error; SII: systemic immune-inflammation index; SMI: Skeletal Muscle Index; WPRT: whole-pelvis RT.

**Table 3:** Multivariable linear regression of predictors of GI symptom score difference.

Predictor			Slope		
	Categories (n)	Adj b ± SE			*p*
Tobacco exposure					0.38
	Non-exposed (17)	Ref			
	Exposed (15)	1.1 ± 1.20			
Constipation history					0.22
	No (23)	Ref			
	Yes (9)	−1.7 ± 1.39			
BMI category					0.02 *
	Normal (5)	Ref			
	Overweight (18)	−1.2 ± 1.63			(0.47)
	Obese (9)	−4.8 ± 1.80			(0.015) *
Previous prostatectomy					0.24
	No (16)	Ref			
	Yes (16)	1.5 ± 1.21			
Concomitant ADT					0.96
	No (3)	Ref			
	Yes (29)	0.14 ± 3.01			
RT field					0.71
	PORT (5)	Ref			
	WPRT (27)	0.9 ± 2.40			
DQI level					0.18
	Low (5)	Ref			
	Moderate (24)	−0.7 ± 1.63			0.67
	High (3)	−4.7 ± 2.69			0.097
SII		0.008 ± 0.003			0.018 *

Adj b: adjusted slope for all other covariates; ADT: androgen deprivation therapy; BMI: body mass index; DQI: Diet Quality Index; PORT: prostate/prostate bed only RT; RT: Radiation Therapy; SE: Standard Error; WPRT: whole-pelvis RT; SII: systemic immune-inflammation index. * *p* < 0.05.

**Table 4:** Multivariable linear regression of predictors of GI symptom score difference (second model).

Predictor		Slope			
	Categories (n)	Adj b ± SE			*p*
BMI category					0.02 *
	Normal (5)	Ref			
	Overweight (18)	−0.21 ± 1.45			(0.89)
	Obese (9)	−3.6 ± 1.58			(0.031) *
DQI level					0.03 *
	Low (5)	Ref			
	Moderate (24)	−1.0 ± 1.44			0.49
	High (3)	−6.0 ± 2.30			0.015 *
SII		0.01 ± 0.002			0.001 **

Adj b: adjusted slope for all other covariates; BMI: body mass index; DQI: Diet Quality Index; SE: Standard Error; SII: systemic immune-inflammation index. * *p* < 0.05, ** *p* < 0.01.

**Table 5:** Prevalence and univariate logistic regression analysis of the variables’ association with grade ≥ 2 GI toxicity (n = 32).

Predictor			≥Grade 2 GI Toxicity		
	Categories (n)	%	OR	(95% CI)	*p*
Tobacco exposure					0.51
	Non-exposed (17)	35.3	1		
	Exposed (15)	46.7	1.60	(0.39; 6.88)	
Alcohol consumption					0.62
	Within recommendations (18)	44.4	1		
	Exceed recommendations (14)	35.7	0.69	(0.16; 2.89)	
Constipation history					0.29
	No (23)	34.8	1		
	Yes (9)	55.6	2.34	(0.49; 12.0)	
BMI category					0.07 '
	Normal (5)	60.0	1		
	Overweight (18)	50.0	0.67	(0.07; 4.99)	(0.69)
	Obese (9)	11.1	0.08	(0.003; 1.03)	(0.08) '
Abdominal waist					0.60
	Low/medium CV risk (13)	46.2	1		
	High CV risk (19)	36.8	0.68	(0.16; 2.89)	
Sarcopenia on CT					0.95
	No (17)	41.2	1		
	Yes (15)	40.0	0.95	(0.23; 3.96)	
Physical activity level					0.77
	Inactive (11)	36.4	1		
	Low to moderately active (10)	50.0	1.75	(0.31; 10.7)	(0.53)
	Active (11)	36.4	1.00	(0.17; 5.88)	(1.00)
SII risk group					0.12 '
	Low risk (20)	30.0	1		
	High risk (12)	58.3	3.27	(0.75; 15.6)	
DQI level					0.04 *
	Low (5)	80.0	1		
	Moderate (24)	37.5	0.15	(0.007; 1.21)	(0.11)
	High (3)	0.0	NA	(NA; NA)	(0.99)
Previous prostatectomy					0.28
	No (16)	31.2	1		
	Yes (16)	50.0	2.20	(0.53; 9.84)	
Concomitant ADT					0.99
	No (3)	0.0	1		
	Yes (29)	44.8	NA	(NA; NA)	
RT field					>0.99
	PORT (5)	0.0	1		
	WPRT (27)	48.1	NA	(NA; NA)	

' *p* < 0.2, * *p* < 0.05, Wald test and global likelihood ratio test for categorical variable with more than two modalities. ADT: androgen deprivation therapy; BMI: body mass index; CI: confidence interval; CT: Computed Tomography; CV: cardio-vascular; DQI: Diet Quality Index; OR: odds ratio; PORT: prostate/prostate bed only RT; RT: Radiation Therapy; SII: systemic immune-inflammation index; WPRT: whole-pelvis RT.

**Table 6:** Variables associated with ≥grade 2 GI toxicity (multivariable logistic regression; reduced model) (n = 32).

	Predictor		≥Grade 2 GI Toxicity		Likelihood Ratio Test
	Categories (n)	%	Adj OR	(95% CI)	*p*
BMI category					0.08
	Normal (5)	60.0	1		
	Overweight (18)	50.0	0.27	(0.02; 2.24)	(0.24)
	Obese (9)	11.1	0.04	(0.0009; 0.57)	(0.02 *)
SII risk group					0.06
	Low risk (20)	30.0	1		
	High risk (12)	58.3	9.75	(0.64; 27.5)	(0.15)
DQI level					0.29
	Low (5)	80.0	1		
	Moderate (24)	37.5	0.19	(0.015; 1.48)	(0.12)
	High (3)	0.0	0.04	(0.0002; 1.27)	(0.07)

* *p* < 0.05; *p*-values between brackets are profile penalized likelihood *p*-values. adj OR: adjusted odds ratio; BMI: body mass index; DQI: Diet Quality Index; SII: systemic immune-inflammation index.

## Data Availability

The data presented in this study are available upon reasonable request from the corresponding author. The data are not publicly available due to patient privacy and ethical restrictions.

## Abbreviations

The following abbreviations are used in this manuscript:

ADT	Androgen deprivation therapy
AIC	Akaike Information Criteria
ANOVA	Analysis of Variance
b	Linear regression slope
BMI	Body mass index
CBCT	Cone-Beam Computed Tomography
CI	Confidence interval
CROs	Clinician-reported outcomes
CT	Computed Tomography
CTCAE	Common Toxicity Criteria for Adverse Events
CTV	Clinical target volume
DQI	Diet Quality Index
ECOG	Eastern Cooperative Oncology Group
EORTC	European Organization for Research and Treatment of Cancer
GI	Gastro-Intestinal
IGRT	Image-guided radiation therapy
IQR	Interquartile range
MIM	Medical Image Merge
OAR	Organ At Risk
OR	Odds ratio
PCa	Prostate cancer
PET	Positron Emission Tomography
PORT	Prostate/prostate bed only radiation therapy
PROs	Patient-reported outcomes
PRV	Planning organ at Risk Volume
PTV	Planning Target Volume
QLQ	Quality of Life Questionnaire
QoL	Quality of life
RT	Radiation Therapy
SD	Standard Deviation
SE	Standard Error
SIB	Simultaneous integrated boost
SII	Systemic immune-inflammation index
SMI	Skeletal Muscle Index
VMAT	Volumetric modulated arc therapy
WPRT	Whole-pelvis radiation therapy
